# Molecular Characteristics and Antibiotic Resistance Profiles of* Klebsiella* Isolates in Mthatha, Eastern Cape Province, South Africa

**DOI:** 10.1155/2017/8486742

**Published:** 2017-01-30

**Authors:** Sandeep Vasaikar, Larry Obi, Isaac Morobe, Mary Bisi-Johnson

**Affiliations:** ^1^Division of Medical Microbiology, Department of Laboratory Medicine and Pathology, Faculty of Health Sciences, Walter Sisulu University, Private Bag X1, Mthatha, Eastern Cape 5117, South Africa; ^2^Academic Affairs, University of Fort Hare, Private Bag X1314, Alice 5700, South Africa; ^3^Department of Biological Sciences, University of Botswana, Mobutu Drive, P.O. Box 80462, Gaborone, Botswana; ^4^Department of Microbiology, Obafemi Awolowo University, Ile-Ife, Osun State, Nigeria

## Abstract

The increase in the incidence of extended-spectrum *β*-lactamase- (ESBL-) producing* Klebsiella* species has become a serious problem worldwide, because of their incrimination in antibiotic resistance. The objective of this study is to investigate the resistance genes responsible for ESBL-producing* Klebsiella *species and carbapenemase-producing* Klebsiella* (CRE) isolated in Mthatha and to study their epidemiology. A prospective, descriptive study of 202 nonrepetitive samples from patients was obtained from Nelson Mandela Academic Hospital. The cultured* Klebsiella* isolates were subjected to antimicrobial susceptibility tests and the polymerase chain reaction of *bla*_CTX-M_, *bla*_TEM_, *bla*_SHV_, *bla*_KPC_, and *bla*_NDM_ genes. Overall* K. pneumoniae *were the majority with 169 (83.7%) species isolates, followed by* K. oxytoca* with 29 (14.4%), while* K. ozaenae* and* Raoultella ornithinolytica* were 2 (0.9%) each. The prevalence of ESBL production in all* Klebsiella* species was 117 (57.9%). ESBL-genotypic resistance is driven in Mthatha by *bla*_SHV_ 121 (77.1%) followed by *bla*_TEM_ 105 (66.9%) and *bla*_CTX-M_ at 89 (56.7%). The most common ESBL genotype combination among the* Klebsiella *was *bla*_TEM_ + *bla*_SHV_ + *bla*_CTX-M_ at 79 (50.3%). There is a steady increase in the rate of ESBL genes in the last five years.

## 1. Introduction

The genus* Klebsiella* belongs to the Enterobacteriaceae family and comprises Gram-negative opportunistic nonmotile pathogens with a mucoid aspect. The gastrointestinal tract serves as a reservoir and is often the latent source for infections [1]. The genus* Klebsiella *is classified into four species:* Klebsiella pneumoniae* (*K. pneumoniae*),* Klebsiella oxytoca *(*K. oxytoca*),* Klebsiella terrigena* (*K. terrigena*), and* Klebsiella planticola *(*K. planticola*), with* K. pneumoniae *consisting of three subspecies,* K. pneumoniae *subsp.* pneumoniae*,* K. pneumoniae *subsp.* ozaenae*, and* K. pneumoniae *subsp.* rhinoscleromatis *[2]*. K. pneumoniae* is an opportunistic microorganism which causes serious diseases such as septicemia, pneumonia, urinary tract infections (UTIs), chronic lung disorders, and nosocomial infections in immunocompromised patients [3]. The emergence of extended-spectrum *β*-lactamase- (ESBL-) producing bacteria, particularly in* K. pneumoniae*, is now a critical concern for the development of therapies against bacterial infection [4]. These strains are resistant to extended-spectrum beta-lactam antibiotics, aminoglycosides, and fluoroquinolones [5]. The currently dominant ESBLs belonging to class A in the Ambler classification are *bla*_TEM_, *bla*_SHV_, and *bla*_CTX-M_ types. The detection of the common ESBL genes such as *bla*_TEM_, *bla*_SHV_, and *bla*_CTX-M_ by molecular methods in the ESBL-producing bacteria and their patterns of antimicrobial resistance can provide useful information about their epidemiology and can aid in developing rational antimicrobial therapy.* K. pneumoniae *carbapenemase (KPC) are Ambler class A plasmid-encoded enzymes that are capable of hydrolyzing all beta-lactam antibiotics, including monobactams, extended-spectrum cephalosporins, and carbapenems. Originally described in 2001, pathogens harbouring these antibiotic resistance enzymes have been reported from the United States of America, France, China, Sweden, Norway, Colombia, and Brazil. Molecular detection of the *bla*_KPC_ gene by polymerase chain reaction (PCR) assay provides laboratories with a means to quickly identify the presence of this important resistance determinant gene [6]. Though many studies have reported the drug resistance of* K. pneumoniae* and* K. oxytoca* worldwide [4, 7–9]. Epidemiology studies on ESBL-producing* K. pneumoniae* in Republic of South Africa (RSA) from different provinces have been reported [10–13], but little is known in the Eastern Cape Province (ECP) about the epidemiology and molecular characteristics of ESBLs. The aim of this study was to investigate the resistance mechanisms to among ESBL-producing different* Klebsiella *species and carbapenemase-producing* Klebsiella* (CRE) isolated in Mthatha and surrounding areas and to study antimicrobial susceptibility to parenteral and oral antimicrobials.

## 2. Materials and Methods

### 2.1. Experimental Design

#### 2.1.1. Ethical Considerations

Ethical approval for the study was granted by the Health Research Ethics and Biosafety Committee of the Walter Sisulu University (WSU) certificate number 022/110 and the Nelson Mandela Academic Hospital Ethics Committee (NMAH), Mthatha, ECP.

#### 2.1.2. Study Design and Setting

**Figure 1:**
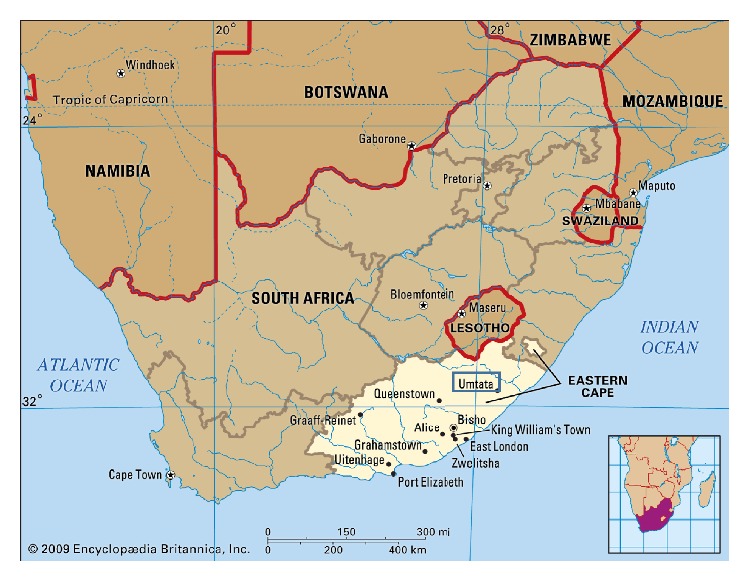
Map of South Africa showing study area, Umtata (now Mthatha), in the province of Eastern Cape (by courtesy of Encyclopaedia Britannica, Inc., copyright 2009; used with permission) [14].

A prospective, descriptive study based on laboratory investigations at the Microbiology Laboratory of the National Health Laboratory Services (NHLS) at NMAH and the Department of Medical Microbiology, Faculty of Health Sciences, WSU was undertaken. In this study 203 nonrepetitive (one per patient) samples from patients were randomly obtained from August 2011 to May 2014.  Figure 1 shows the specimen catchment area, that is, Mthatha and surrounding clinics. Mthatha (formerly Umtata) is the main town of the King Sabata Dalindyebo Local Municipality in the Oliver Reginald Tambo District of the ECP in South Africa. Study areas and health facilities in Mthatha and surrounding areas were primary health centres/clinics, secondary district hospitals, and a tertiary/academic hospital.

#### 2.1.3. Specimens

Nonduplicate, randomly selected* Klebsiella* isolates were collected from Mthatha and surrounding-area clinics. Specimens included blood culture and catheter tips, swabs from abscesses, eye, ear, and vagina, sputum and throat swabs, urine, and sterile fluids (plural fluid, synovial fluid, etc.). Demographic data of the patients recorded were date of specimen collection, age, gender, specimen, tests ordered, and hospital/clinic and provisional diagnosis.

### 2.2. Microbiologic Methods

All samples were routinely cultured on MacConkey and blood agar plates. Blood and sputum were also cultured on chocolate agar. All suspected colonies were identified by gram staining, colony characteristics, motility, and so forth. Strains were identified to the species level with bioMérieux API20E and confirmed by Siemens MicroScan Negative ID panel Type 2. MICs were determined using MicroScan dehydrated broth microdilution panel negative MIC Type 37 (Siemens Medical Solutions Diagnostics, West Sacramento, CA), following the manufacturer's guidelines and Clinical Laboratory Standards Institute (CLSI) [15]. MICs were interpreted following CLSI guidelines, including the new clinical breakpoints published in 2010 for carbapenems [16]. ESBL detection: phenotypic—the ESBL detection was done as was recommended by the CLSI confirmatory procedure, by using cefotaxime (30 *μ*g) and ceftazidime (30 *μ*g) discs alone and in combination with clavulanic acid discs.* K. pneumoniae* (ATCC-700603) were used as the controls throughout the study [17]. The ESBL production was confirmed by MicroScan MIC 37 panel using combination of cefotaxime/K clavulanate (Cft/CA) and ceftazidime/K clavulanate (Caz/CA) [18].

### 2.3. Molecular ESBL Detection by rPCR

#### 2.3.1. DNA Extraction

DNA extraction was done using Roche MagNA Pure Bacteria Lysis Buffer, MagNA Pure Compact Nucleic Acid Isolation Kit 1 in MagNA Pure Compact System (Roche Applied Science, Indianapolis).

#### 2.3.2. Real-Time PCR for *bla*_CTX-M_, *bla*_TEM_, *bla*_SHV_, *bla*_NDM_, and *bla*_KPC_

Real-time polymerase chain reaction (rPCR) was carried out in the LightCycler 2.0 instrument (Roche Applied Science, Germany) using LightCycler 480 Probes Master kit (Roche Diagnostics, USA). The *bla*_CTX-M_, *bla*_TEM_, and *bla*_SHV_ for ESBL and *bla*_NDM_ and *bla*_KPC_ genes for CRE were amplified by singleplex rPCR using the primers shown in Table 1. Primers were designed by TIB-Molbiol (Berlin, Germany) based on primers used by Turton et al. [19]. rPCR assay was performed in a 32 carousels using 20 *μ*L capillaries with each capillary containing a total volume of 20 *μ*L including 2 *μ*L of LightCycler FastStart DNA Master HybProbe, 2 *μ*L of primers (0.5 mM for each forward and reverse), 2.4 *μ*L of MgCl_2_, 2 *μ*L of extracted DNA, and water to make up the volume of 20 *μ*L. DNA amplification was carried out using preincubation step at 95°C for 30 s, followed by 45 cycles of amplification with denaturation at 95°C for 30 s, annealing and extension at 60°C for 1 minute without the third step, and then a single cycle of cooling. Absolute quantification was carried out using the LightCycler software 4.05.

### 2.4. Statistical Analysis

Our data was entered into a database using SPSS 23 for Windows (SPSS Inc., Chicago, IL). The primary analysis involved using chi-square test in order to look for association between dependable variable (ESBL production) with covariable, for example, type of hospital/clinic, gender, and type of specimens. Significant variables were included in binary logistical regression to calculate the odds ratio and 95% confidence interval. All statistical testing was two tailed and statistical significance was defined as ≤0.05. Comparison of ESBL and non-ESBL groups was done using Epidat 3.1 software.

## 3. Results

During the study period, a total of 202* Klebsiella* species were isolated from a range of clinical specimens of patients hospitalized in various wards of NMAH in Mthatha and surrounding areas with secondary hospitals and clinics.* K. pneumoniae* were the majority 169 (83.7%) species isolated followed by* K. oxytoca *29 (14.4%) while* K. ozaenae* and* R. ornithinolytica* were 2 (0.9%). The prevalence of ESBL production in all* Klebsiella* species was 122 (60.4%) while ESBL-producing* K. pneumoniae* were 117 (69.2%) followed by* K. oxytoca *5 (17.9%) and both* K. ozaenae* and* R. ornithinolytica* were ESBL negative. Female population was slightly more 106 (52.5%) than males 96 (47.5%).

In the multivariate analysis (backward logistic regression), using age group from day 1 to 5 years and tertiary level of hospitalization were found to be independent risk factors for infection due to ESBL* Klebsiella* species (Table 2).

High antibiotic resistance in decreasing order was observed in amp/sulbactam, mezlocillin 167 (82.7%), piperacillin 160 (79.2%), trimeth/sulfa 143 (70.8%), cefazolin 139 (68.8%), cefepime 130 (64.4%), cefuroxime 129 (63.9%), cefpodoxime 127 (62.9%), aztreonam 126 (62.4%), ceftazidime 124 (61.4%), and tobramycin 108 (53.5%).

Tables 4 and 5 show rPCR data, *bla*_CTX-M_, *bla*_TEM_, *bla*_SHV_, *bla*_NDM_, and *bla*_KPC_ rPCR, and LightCycler 2.0 results; real-time PCR was done on 157 specimens.

rPCR was done on 157 specimens which were 103 (65.6%) of ESBL positive* K. pneumoniae* and* K. oxytoca* and 54 (34.4%) ESBL negative* K. pneumoniae*,* K. oxytoca*,* K. ozaenae*, and* R. ornithinolytica*. In rPCR the most common genotype was *bla*_SHV_ 121 (77.1%) followed by *bla*_TEM_ 105 (66.9%) and *bla*_CTX-M_ at 89 (56.7%) as last.

As seen in Table 5 there is increase trend of ESBL genotypes over four years of study from 2011 to 2014 except in year 2013. The carbapenemase genes *bla*_NDM_ and *bla*_KPC_ were not detected.

## 4. Discussion

The emergence of plasmid mediated ESBLs among the members of* Klebsiella *has increased worldwide. The incidence of ESBLs in the different parts of South Africa has been reported from 36.1% to be as high as 68.3% (Gauteng, Western Cape, KwaZulu-Natal, Free State, and Limpopo) [11–13]. ESBL in* K. pneumoniae* showed rate of 62% from seven public sector hospitals in 2010 [20]. We reported 57.9% (117) ESBL producers, which is less than the above-mentioned South African provinces. In all of the above-mentioned studies ECP was not represented; this could be reason for slightly less rate of ESBLs in this area. In Africa ESBLs rate in different countries have increased from 11.7 to 77.8% among* K. pneumoniae* (study periods: 1999–2005 and 2010) [7, 21]. Our ESBL rate of 57.9% is within this range, but it is on higher side. It was interesting to note that specific ESBLs appeared to be unique to a certain country or region. Though the prevalence of ESBLs has been recognized in various parts of the country, there is only limited data on its genotypes in this part of South Africa.

High antibiotic resistance in decreasing order was observed in penicillins, cephalosporins, folate pathway inhibitors, monobactams, and aminoglycosides. Percentage of low resistance was seen in carbapenems, aminoglycosides (only amikacin), glycylcyclines (tigecycline), cephamycins (cefoxitin), quinolone (levofloxacin), phosphonic acids (fosfomycin), antipseudomonal penicillins + *β*-lactamase inhibitor (pip/tazo), and fluoroquinolones (ciprofloxacin) which can be considered for treatment of* Klebsiella* species in Mthatha. Although resistance of* K. pneumoniae* to ceftazidime is a useful marker of presence of ESBLs, some types of ESBL-producing organisms appear susceptible to ceftazidime according to standard methods, and ceftazidime resistance may be due to mechanisms other than ESBL production. We detected 61.4% resistance to ceftazidime by MIC method while 57.9% (117)* Klebsiella* were ESBL producers by the CLSI confirmatory test which correlates well. Antibiotic resistance to cefotaxime, ceftazidime, and cefepime was 125 (61.9%), 124 (61.4%), and 130 (64.4%), respectively, which is almost identical. This suggests that there are multiple copies of several ESBL genes in the sample, which is supported by the genotypic results in which 66.2% specimens had two or more ESBL genes (Table 3). We observed that nearly two-thirds of the* Klebsiella* ESBL isolates were also resistant to at least 2 useful non-*β* lactam antibiotics used to treat UTI, such as ciprofloxacin, trimethoprim-sulfamethoxazole, and gentamicin. Similar observations have been made by other investigators [22].

In this study the most common genotype was *bla*_SHV_ 118 (75.2%) followed by *bla*_TEM_ 99 (63.1%) and *bla*_CTX-M_ 89 (56.7%) as last. These genotypes were found in majority of ESBL positive* Klebsiella* species as compared to ESBL negative* Klebsiella*. Our study finding is similar to the study done from Italy in which *bla*_SHV_ was found to be the dominant ESBL genotype and also from Spain [22, 23]. ESBL genotype varies in different parts of the world; *bla*_CTX-M_ is the most prevalent gene in high proportion of samples from different parts of the world and also Morocco [7, 9]. In Africa *bla*_CTX-M15_ is the most prevalent gene in a high proportion of the samples, disregarding country. It is usually combined with other types of *bla*_CTX-M_, *bla*_TEM_, and *bla*_SHV_ genes [7]. In other parts of the world, for example, Canada [8] and Turkey [24], *bla*_TEM_ was the most common ESBL genotype. The most common ESBL genotype combination among the* Klebsiella* species (especially in* K. pneumoniae*) was combination of *bla*_TEM_ + *bla*_SHV_ + *bla*_CTX-M_ which is similar to a previous report from South Africa [12]. The second most common genotype combination was the combination of *bla*_TEM_ + *bla*_SHV_ which is similar to the study from Japan [4]. We found increased trend of* Klebsiella* ESBL genotypes over 4 years; this trend is seen in studies from different parts of the world, Japan and Canada [4, 8].

CRE has become an international health issue and poses a major threat to the viability of currently available antibiotics. First KPC was reported from South Africa in August 2011 from private hospital in Gauteng [25]. In our study we observed resistance to carbapenems by phenotypic test as ertapenem 3.5% while meropenem and imipenem were all susceptible. But all specimens were negative for *bla*_KPC_ and *bla*_NDM_ by genotypic test; this could be due to other resistance mechanisms such as newer CRE genes Verona Integron-Mediated Metallo-*β*-Lactamase (VIM), Imipenemase Metallo-Beta-Lactamase (IMP), or class D OXA-type enzymes [26].

In multivariate analysis we found age group 1–5 years (OR 2.32 [CI 1.20–4.52]) and tertiary health centre (OR 5.96 [CI 2.21–16.03]) were risk factors for developing ESBL. In ESBLs we found statistically significant (*P* < 0.005) antibiotic resistance in amox/clav, ampicillin, cefotaxime, cefuroxime, chloramphenicol, ciprofloxacin, gentamicin, levofloxacin, moxifloxacin, pip/tazo, tetracycline, tobramycin, and trimeth/sulfa.

## 5. Conclusion

This is the first report of molecular characteristics and antibiotic resistance profiles of* Klebsiella* isolates from clinical samples of patients in Mthatha, ECP, South Africa. The majority of* Klebsiella* species in our area are* K. pneumoniae* followed by* K. oxytoca* and less than 1% of* K. ozaenae* and* R. ornithinolytica*. This study reveals high ESBL rate in* Klebsiella* species especially in* K. pneumoniae *in this area. We also identified ESBL-producing three genes of *bla*_TEM_, *bla*_SHV_, and *bla*_CTX-M_ by rPCR and our antibiotic resistance in* Klebsiella* species in Mthatha which is driven by combination of *bla*_TEM_ + *bla*_SHV_ + *bla*_CTX-M_ and the most common genotype was *bla*_SHV_ followed by *bla*_TEM_ and *bla*_CTX-M_ as last. For treatment of drug resistant* Klebsiella* choice of antibiotics in decreasing order is carbapenems, amikacin, tigecycline, cefoxitin, levofloxacin, pip/tazo, ciprofloxacin, and fosfomycin for UTI. Fortunately we did not detect CRE-forming* Klebsiella* in Mthatha. There is steady increase in rate of ESBL genes in the last five years; therefore continuous surveillance is essential to monitor the ESBL-producing* Klebsiella *in hospitals and community and also for CRE.

## Figures and Tables

**Table 1:** Primer sequences used for *bla*_CTX-M_, *bla*_TEM_, *bla*_SHV_, *bla*_KPC_, and *bla*_NDM_ genes detection.

Target gene	Primer sequences (5′-3′)	Temp in °C	Target size bp
CTX-M forward primer	ATG TGC AGY ACC AGT AAR GTK ATG GC	58.7	336 bp
CTX-M reverse primer	ATC ACK CGG RTC GCC NGG RAT	59.3	
CTX-M1 probe	FAM-CCC gAC AgC Tgg gAg ACg AAA CgT	70.2	
CTX-M2 probe (gr 2)	YAK-CAggTgCTTATCgCTCTCgCTCTgTT	66.3	
CTX-M9 all/1 probe	LC640-Cg AC AAT ACNgCC ATg AA	41.0	
CTX-M9 probe	LC610-CTggATCgCACTgAACCTACgCTgA	53.0	
TEM forward primer	AAG TTC TGC TAT GTG CGG TA	59.7	180 bp
TEM reverse primer	TGT TAT CAC TCA TGG TTA TGG CAG C	59.5	
SHV forward primer	CAG GAT CTG GTG GAC TAC T	57.2	195 bp
SHV reverse primer	GTC AAG GCG GGT GAC GTT	59.3	
SHV-A primer	AAG GCG GGT GAC GTT GTC	59.3	
SHV-S primer	CCG GTC AGC GAA AAA CAC	57.0	
SHV probe	Cy5-TCT GGC GCA AAA AGG CAG TCA-BBQ	64.5	
NDM forward primer	GAC CGC CCA GAT CCT CAA	57.5	51 bp
NDM reverse primer	CGC GAC CGG CAG GTT	60.2	
NDM probe	FAM-TGG ATC AAG CA+GGA+GAT-BBQ	48.8	
KPC forward primer	GGC CGC CGT GCA ATA C	58,6	61 bp
KPC reverse primer	GCC CAA CTC CTT CA	59,6	
KPC probe	Cy5-TGA TAA CGC GCG CAA TTT GT-BBQ	68.9	

Relevant positive strains for *Klebsiella *used as positive control in rPCR: CTX-M (group 1)—*E. coli* ATCC 25922, CTX-M (all groups except group 1)—CAP2006-D17*∗*, TEM—*K. pneumoniae *ATCC 51503, SHV—*K. pneumoniae* ATCC 700603, KPC—*K. pneumoniae *ATCC BAA 1705, NDM—*K. pneumoniae *ATCC BAA 21246. Obtained from NICD (National Institute of Communicable Diseases, Johannesburg, South Africa).

**(a):** 

Quantitative variable	ESBL	NIL	OR (95% CI)	*P value*
	Mean ± SD	Mean ± SD		
Age (groups 0–5)	22.78 (22.77)	30.9 (22.7)	2.32 (1.20–4.52)	**0.012**

**(b):** 

Variable	ESBL	NIL	OR (95% CI)	*P value*
	Number (%)	Number (%)		
Gender	****			
Male	59 (60.8)	38 (39.2)	1.18 (0.68–2.06)	0.551
Female	58 (55.2)	47 (44.8)		
Type of hospital				
Primary^*∗*^	3 (21.4)	11 (78.6)	—	—
Secondary	41 (47.1)	46 (52.9)	2.06 (0.76–5.57)	0.153
Tertiary	73 (72.3)	28 (27.7)	5.96 (2.21–16.03)	**0.000**

^*∗*^Reported as resistant due to ESBL production.

**Table 3:** Percentage of resistance (included intermediate resistance for statistical analysis) in ESBL and non-ESBL *Klebsiella *(*N* = 202).

Antibiotic	Total (*n* = 202)	ESBL (*n* = 117)	NIL (*n* = 85)	OR (95% CI)	*P value* ^a^
	Number (%)	Number (%)	Number (%)		
Amikacin	10 (5.0)	5 (4.3)	5 (5.9)	0.71 (0.20–2.55)	0,604
Amox/K clav	111 (55.0)	98 (83.3)	13 (15.3)	28.6 (13.25–61.59)	**0,000**
Ampicillin	137 (67.8)	115 (98.3)	22 (25.9)	162.0 (36.88–711.93)	**0,000**
Amp/sulbactam^*∗*^	201 (99.5)	117 (100.0)	84 (98.8)	—	1,000
Aztreonam^*∗*^	126 (62.4)	117 (100.0)	9 (10.6)	—	0,996
Cefazolin^*∗*^	139 (68.8)	117 (100.0)	22 (25.9)	—	0,996
Cefepime^*∗*^	130 (64.4)	117 (100.0)	13 (15.3)	—	0,996
Cefotaxime	124 (61.4)	117 (100.0)	7 (8.2)	116.5 (136.9–9105.9)	**0,000**
Cefoxitin	18 (8.9)	10 (8.5)	8 (9.4)	0.90 (0.34–2.38)	0,831
Cefpodoxime^*∗*^	127 (62.9)	117 (100.0)	10 (11.8)	—	0,996
Ceftazidime^*∗*^	124 (61.4)	117 (100.0)	7 (8.2)	—	0,996
Cefuroxime	129 (63.9)	117 (99.1)	13 (15.3)	642.5 (82.29–5016.08)	**0,000**
Chloramphenicol	71 (35.1)	53 (45.3)	18 (21.2)	3.25 (1.71–6.19)	**0,000**
Ciprofloxacin	60 (29.7)	55 (47.0)	5 (5.9)	14.19 (5.36–37.58)	**0,000**
Ertapenem	7 (3.5)	4 (3.4)	3 (3.5)	0.97 (0.21–4.44)	0,966
Fosfomycin	26 (12.9)	15 (12.8)	11 (12.9)	0.97 (0.42–2.25)	0,954
Gentamicin	103 (51.0)	92 (78.6)	11 (12.9)	24.76 (11.44–53.60)	**0,000**
Imipenem	0 (0.0)	0 (0.0)	0 (0.0)	—	—
Levofloxacin	23 (11.4)	19 (16.2)	4 (4.7)	3.88 (1.27–11.86)	**0,018**
Meropenem	0 (0.0)	0 (0.0)	0 (0.0)	—	—
Mezlocillin^*∗*^	167 (82.7)	117 (100.0)	50 (58.8)	—	0,997
Moxifloxacin	77 (38.1)	65 (55.6)	12 (14.1)	8.43 (4.10–17.32)	**0,000**
Nitrofurantoin^Φ^	1 (0.5)	0 (0.0)	1 (1.2)	—	1,000
Norfloxacin^Φ^	3 (1.5)	3 (2.6)	0 (0.0)	—	0,999
Pip/tazo	31 (15.3)	28 (23.9)	3 (3.5)	8.60 (2.52–29.36)	**0,001**
Piperacillin^*∗*^	160 (79.2)	117 (100.0)	43 (50.6)	—	0,997
Tetracycline	57 (28.2)	44 (37.6)	13 (15.3)	3.29 (1.64–6.63)	**0,001**
Tigecycline	17 (8.4)	9 (7.7)	8 (9.4)	0.80 (0.30–2.17)	0,664
Tobramycin	108 (53.5)	102 (87.2)	6 (7.1)	88.40 (32.79–238.28)	**0,000**
Trimeth/sulfa	143 (70.8)	115 (98.3)	28 (32.9)	117.05 (26.93–508.72)	**0,000**

^a^Exact *P* values were determined by the *χ*^2^ test. For statistical analysis, the response of isolates to antibiotics was categorized as susceptible and nonsusceptible (consisting of intermediate and resistant groups). A *P* value < 0.05 was considered statistically significant. ^*∗*^Reported as resistance due to ESBL production according to confirmation by Autoscan MIC37 panel. Φ: used only in urinary tract infections.

**Table 4:** Extended-spectrum *β*-lactamase (ESBL) genotypes in *Klebsiella* strains.

Positive by PCR for ESBL genes	Number amplified			
	*K. pneumoniae *(*n* = 139)	*K. oxytoca* (*n* = 16)	*R. ornithinolytica *and* K. ozaenae* (*n* = 2)	Total (*N* = 157)
*(A) Single ESBL gene*	*28*	*2*	*2*	*32 (20.4%)*
*bla*_TEM_ only	5	2	1	8 (5.1%)
*bla*_SHV_ only	22	0	1	23 (14.7%)
*bla*_CTX-M_ only	1	0	0	1 (0.6%)
*bla*_KPC_ only^*∗*^	0	0	0	0
*bla*_NDM_ only^*∗*^	0	0	0	0
*(B) Two or more ESBL genes*	*100*	*3*	*0*	*103 (65.6%)*
*bla*_CTX-M_ + *bla*_TEM_	4	0	0	4 (2.6%)
*bla*_CTX-M_ + *bla*_SHV_	6	0	0	6 (3.8%)
*bla*_TEM_ + *bla*_SHV_	12	2	0	14 (8.9%)
*bla*_TEM_ + *bla*_SHV_ + *bla*_CTX-M_	78	1	0	79 (50.3%)
*bla*_KPC_ + *bla*_NDM_^*∗*^	0	0	0	0

^*∗*^Done in 52 selective ESBL positive isolates.

**Table 5:** Genotypes of *Klebsiella bla*_TEM_, *bla*_SHV_, and *bla*_CTX-M_ from 2011 to 2014.

Genotypes	2011	2012	2013	2014	*Total* (*N* = 157)
*bla*_TEM_ positive alone or in combination	23/34 (67.7%)	63/98 (67.3%)	6/8 (75%)	13/17 (76.5%)	*105 (66.9%)*
*bla*_SHV_ positive alone or in combination	19/34 (55.9%)	79/98 (80.6%)	8/8 (100%)	15/17 (88.2%)	*121 (77.1%)*
*bla*_CTX-M_ positive alone or in combination	17/34 (50%)	54/98 (55.1%)	6/8 (75%)	12/17 (70.6%)	*89 (56.7%)*
